# An Eco-Friendly Manner to Prepare Superwetting Melamine Sponges with Switchable Wettability for the Separation of Oil/Water Mixtures and Emulsions

**DOI:** 10.3390/polym16050693

**Published:** 2024-03-03

**Authors:** Guyita Berako Belachew, Chien-Chieh Hu, Yan-Yu Chang, Chih-Feng Wang, Wei-Song Hung, Jem-Kun Chen, Juin-Yih Lai

**Affiliations:** 1 Graduate Institute of Applied Science and Technology, Advanced Membrane Materials Research Center, National Taiwan University of Science and Technology, Taipei 106, Taiwan; barakobelachew@gmail.com (G.B.B.); cchu@mail.ntust.edu.tw (C.-C.H.); wshung@mail.ntust.edu.tw (W.-S.H.); jylai@mail.ntust.edu.tw (J.-Y.L.); 2 Department of Materials Science and Engineering, I-Shou University, Kaohsiung 840, Taiwan; yann940956@gmail.com; 3 Institute of Advanced Semiconductor Packaging and Testing, National Sun Yat-Sen University, Kaohsiung 804, Taiwan; 4 Department of Materials Science and Engineering, National Taiwan University of Science and Technology, Taipei 106, Taiwan; jkchen@mail.ntust.edu.tw; 5 R&D Center for Membrane Technology, Chung Yuan Christian University, Taoyuan 320, Taiwan; 6 Department of Chemical Engineering and Materials Science, Yuan Ze University, Taoyuan 320, Taiwan

**Keywords:** melamine sponge, sodium dodecanoate, switchable wettability, oil/water mixtures separation, emulsion separation

## Abstract

Oil/water separation processes have garnered significant global attention due to the quick growth in industrial development, recurring chemical leakages, and oil spills. Hence, there is a significant demand for the development of inexpensive superwetting materials in an eco-friendly manner to separate oil/water mixtures and emulsions. In this study, a superwetting melamine sponge (SMS) with switchable wettabilities was prepared by modifying melamine sponge (MS) with sodium dodecanoate. The as-prepared SMS exhibited superhydrophobicity, superoleophilicity, underwater superoleophobicity, and underoil superhydrophobicity. The SMS can be utilized in treating both light and heavy oil/water mixtures through the prewetting process. It demonstrated fast permeation fluxes (reaching 108,600 L m^−2^ h^−1^ for a light oil/water mixture and 147,700 L m^−2^ h^−1^ for a heavy oil/water mixture) and exhibited good separation efficiency (exceeding 99.56%). The compressed SMS was employed in separating surfactant-stabilized water-in-oil emulsions (SWOEs), as well as surfactant-stabilized oil-in-water emulsions (SOWEs), giving high permeation fluxes (reaching 7210 and 5054 L m^−2^ h^−1^, respectively). The oil purity for SWOEs’ filtrates surpassed 99.98 wt% and the separation efficiencies of SOWEs exceeded 98.84%. Owing to their remarkable capability for separating oil/water mixtures and emulsions, eco-friendly fabrication method, and feasibility for large-scale production, our SMS has a promising potential for practical applications.

## 1. Introduction

Oil spills, municipal sewage, and industrial oily wastewater discharge pose a significant hazard to the aquatic environment, and consequently the global ecosystem as well. Therefore, the separation of oil and water has evolved into an expanding and swiftly escalating issue within both the research community and the industrial sector [1,2,3,4,5]. Oil and water mixtures include immiscible oil–water mixture and stabilized emulsions with droplet diameters less than 20 μm. Hence, emulsified oil–water mixtures are harder to separate owing to their stability, and greater focus should be made on innovating inexpensive, reliable, and highly efficient methods to address this issue [6,7,8]. Conventional oil–water separation systems face challenges such as high energy consumption and membrane fouling. Furthermore, a single membrane may not be able to effectively treat all kinds of oil–water mixtures [8,9].

Recently, the utilization of superwetting materials in membrane adsorption and filtration strategies for oil–water separation has garnered considerable attention from researchers due to their potential to surpass traditional methods in terms of cost-effectiveness, efficiency, eco-friendliness, and diverse industrial applications [10,11,12]. Wettability is characterized by both surface chemistry and the geometrical structure of the materials. The chemical composition determines the interaction between the liquid and the solid at the molecular level, while the surface morphology, including features like roughness and texture, can affect how the liquid spreads on the surface [13,14].

Having an appropriate membrane that would not be blocked by a phase that is not permeating is also crucial for achieving greater separation performance. Inspired by fish scale, superhydrophilic/underwater superoleophobic membranes that selectively allow the permeation of water and hindering oil, have been created and developed for various oil–water separation applications [15,16], and such membranes exhibit antifouling properties [17,18]. Accordingly, many super-hydrophilic and underwater super-oleophobic materials have been fabricated from TiO_2_ nanoparticle sprayed meshes [19], aloe vera mucilage coated fabrics [20], poly(vinyl alcohol) hydrogel coated filter paper [21], graphene oxide modified meshes [22], polyacrylamide hydrogel coated meshes [23], and porous nitrocellulose membranes [24]. However, membranes designed for oil blocking are not appropriate to be employed in the separation of heavy oil–water mixtures since heavy oil creates a barrier film on the membrane preventing water permeation.

Materials endowed with superhydrophobicity and superoleophilicity, often referred to as “water blocking” materials, present an ideal solution for the separation of heavy oil and water mixture [10,25]. Accordingly, various superhydrophobic/superoleophilic materials have emerged such as polytetrafluoroethylene (PTFE) coated stainless steel meshes [26], polyaniline and fluorinated alkyl silane modified fabrics [27], poly(vinyl phenol) and phenylene bisoxazoline modified cotton materials [28], Cu(OH)_2_ and N-dodecylmercaptain modified copper foams [29], and perfluorodecyltriethoxysilane and ZnO coated paper [30]. Although fluoropolymers are suitably preferred to lower the surface free energy of the materials, they are expensive and are not environmentally friendly. Additionally, superhydrophobic/superoleophilic materials pose challenges when it comes to selective filtration of water in light oil mixtures since water creates a barrier film between the light oil and the membrane.

In order to address the limitations observed in previously reported materials, developing materials that possess switchable superwetting features for the separation of oil and water, which are in high demand, proves highly advantageous [10,31,32,33]. Membranes which exhibit underwater superoleophobic and underoil superhydrophobic properties can realize the separation of different immiscible oil–water mixtures and oil–water emulsions. Zhang et al. fabricated an underwater superoleophobic and underoil superhydrophobic membrane from a dopamine and polyethyleneimine-modified iron mesh, and the as-prepared mesh displayed outstanding antifouling properties, and had high separation efficiency [34]. Kong et al. successfully fabricated a hygro-responsive membrane by employing liquid-phase deposition of TiO_2_ with perfluorooctanoic acid, which demonstrated high efficiency in separating various oil–water mixtures [35]. Similarly, Chen et al. employed simple methods to create fabrics with underwater superoleophobic and underoil superhydrophobic properties, that were proven effective in separating versatile oil–water mixtures and oil–water emulsions [36]. Gao et al. fabricated a dually prewetted carbon black membrane capable of continuously separating multiphase emulsions with high permeation flux and separation efficiency [37]. Yang et al. developed a superwetting copper-coated fabric that has a wettability that can be switched, enabling a remarkably efficient oil–water separation [38].

However, the above-mentioned materials’ use for large-scale applications has been constrained, though, by the use of hazardous chemicals, complicated fabrication processes, and secondary environmental pollution. As a result, a straightforward, environmentally friendly, and effective method for creating an underwater superoleophobic and underoil superhydrophobic membrane that can be utilized to realize the separation of surfactant-stabilized oil–water emulsions and versatile oil–water mixtures needs to be developed. Commercially available 3D porous polymeric sponges are widely used for oil–water separations due to their ultralight weight, low cost, mechanical strength, high flexibility, and large-scale production [39,40,41]. Among these are melamine-based sponges that have outstanding fire-retardant behavior and high-temperature resistance because of their high nitrogen content [42,43]. One of the most commonly known and commercially available melamine sponges is the “Mr. Clean Magic Eraser”, renowned for its versatility as a flame retardant, noise reducer, and insulation for pipes and ductwork. However, studies on melamine sponge-based materials with both underwater superoleophobic and underoil superhydrophobic properties are rare. These materials can be employed for separation and absorption of organic contaminants from water including water-in-oil emulsion and oil-in water emulsion separation.

In this study, the pristine melamine sponge (MS) was transformed into a superwetting material with switchable wettabilities that demonstrated underwater superoleophobicity and underoil superhydrophobicity using a simple and eco-friendly dip coating method without employing any organic solvents. The fabricated MS mimics the “lotus effect”, exhibiting in-air superhydrophobicity and underoil superhydrophobicity states, and also mimics the “fish-scale effect”, demonstrating in-air superoleophilicity and underwater superoleophobicity states [14,15]. Therefore, the as-prepared MS sponge can be used for a variety of oil/water separation purposes, including separating oil-in-water and water-in-oil emulsions, as well as for on-demand separation of immiscible light oil/water and heavy oil/water mixtures.

## 2. Materials and Methods

### 2.1. Materials

Comprehensive details regarding the materials can be found in the Supporting Information section.

### 2.2. Preparation of Superwetting Melamine Sponge (SMS) with Switchable Wettability

To prepare the superwetting melamine sponge (SMS) with switchable wettability, firstly, the surface impurities of the pristine melamine sponge were removed through a washing process employing water and ethanol, followed by drying at 60 °C for an hour prior to use. Then, 0.5 M HCl and 0.05 M sodium dodecanoate aqueous solutions were prepared. The cleaned MS was soaked in the HCl_(aq)_ for 30 min to enhance acid protonation, followed by repeated washing with water. The protonated melamine sponge was immersed in 0.05 M sodium dodecanoate aqueous solution for a duration of 10 min and was subsequently rinsed with water numerous times. The modified sponge was finally rinsed with ethanol and subsequently dried in an oven at 60 °C for a period of 3 h.

### 2.3. Preparation of Various Emulsions

The surfactant-stabilized water-in-oil emulsions (SWOEs) were formulated by adding Span 80 (0.03 g) to various kinds of organic solvents or oils (100 mL), followed by the addition of water (1 mL) into the solution. The mixture was then subjected to a 3 h stirring process. Surfactant-stabilized oil-in-water emulsions (SOWEs) were formulated by dissolving Tween 80 (0.03 g) in water (100 mL), followed by the addition of organic solvents or oils (2 mL) into the solution, which was subjected to stirring for 3 h.

### 2.4. Oil/Water Mixtures Separation Experiments

In assessing the oil/water separation capability of our fabricated material, the SMS was fixed in a glass funnel and prewetted with either water or oil. Subsequently, a 1:1 volumetric ratio mixture of oil and water was then poured onto the prewetted SMS.

### 2.5. Emulsions Separation Experiments

Emulsion separation experiments were carried out by placing compressed SMS into a glass funnel and prewetting it with oil or water for separating SWOEs or SOWEs, respectively. The separation experiment was undertaken by gravity. To evaluate the flux, the permeation volume of liquid passing through a unit area per unit time for the 50 mL emulsion was calculated.

To calculate the separation efficiency for oil/water mixtures and emulsions the following equation was used:

(1) $$separation\;efficiency=\left(1-\frac{C_{p}}{C_{o}}\right)\times 100\%$$

where *C_o_* represents the water (or oil) content in the feed oil/water mixtures or emulsions, and *C_p_* represents the corresponding filtrate content.

### 2.6. Instruments and Characterization

Comprehensive details regarding the characterizations and the instruments employed can be found in the Supporting Information section.

## 3. Results

### 3.1. Preparation, Morphological Analysis and Surface Chemical Compositions of Melamine Sponges

**Figure 1:**
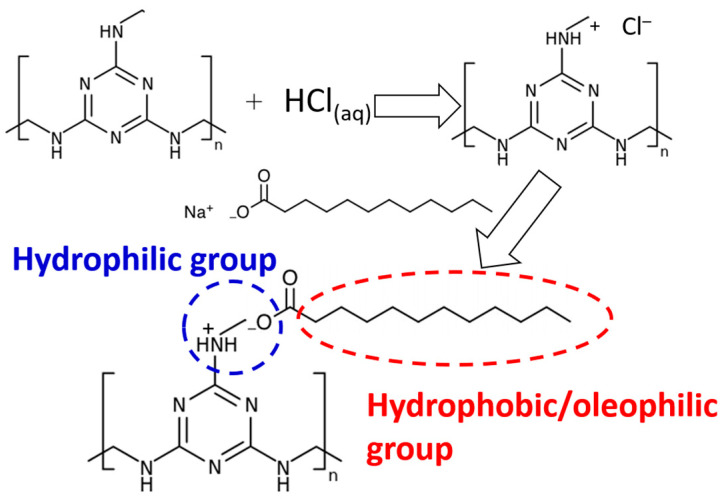
Illustration of the preparation process of the superwetting melamine sponge (SMS).

MS, with its high porosity and good mechanical properties, has been commercialized as an effective abrasive cleaner. In this research, we prepared a superwetting melamine sponge (SMS) possessing switchable wettabilities through an eco-friendly procedure without using any organic solvents. As demonstrated in Figure 1, the pristine MS is immersed in an acidic medium to form protonated MS [44,45]. Then, the protonated MS was treated with a sodium dodecanoate aqueous solution to undergo a counterion exchange reaction [46,47] to have the desired wettability. The combination of the long carbon chain (hydrophobic/oleophilic groups), together with carboxyl and quaternary ammonium groups (hydrophilic groups) on the surface of SMS resulted in switchable superwetting properties.

**Figure 2:**
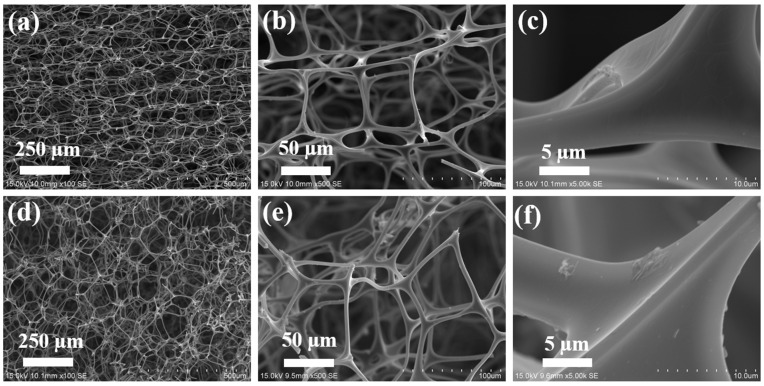
SEM micrographs of (**a**–**c**) pristine MS and (**d**–**f**) SMS.

**Figure 3:**
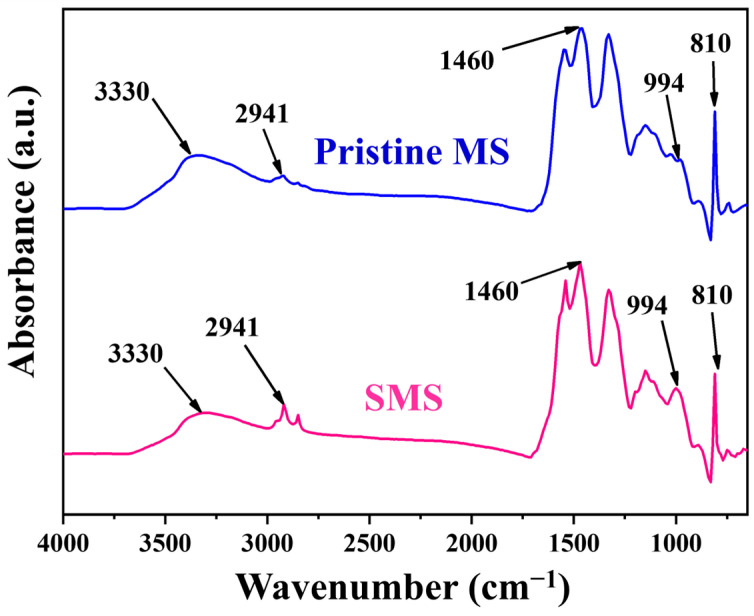
FTIR spectra of the pristine MS and SMS.

Scanning electron microscopy (SEM) was employed to investigate the surface microstructures of the pristine MS and the SMS. The pristine MS showed open-cell porous microstructures (Figure 2a,b) and smooth surface morphologies (Figure 2c). The SEM images indicated that the morphology of SMS closely resembled that of the pristine MS (Figure 2d–f). Figure 3 displays the Fourier transform infrared (FTIR) spectra of the pristine MS and SMS. The broader band at about 3330 cm ^−1^ could be attributed to the N-H secondary amine stretching vibrations. Both MS and SMS showed characteristic peaks at about 1530 cm^−1^, 1460 cm^−1^ and 1323 cm ^−1^ that could correspond to C=N ring stretching vibrations and C-H bending vibrations, respectively. Moreover, the peaks at about 2941 cm^−1^, 994 cm^−1^, and 810 cm^−1^ represent the C-H stretching vibrations, C-H bending vibrations, and triazine ring bending vibrations, respectively [48,49,50]. As can be observed from Figure 3, the increase in the intensity of both C-H stretching and bending vibrations in the SMS sponge spectra confirms that the dodecanoate was successfully incorporated onto the MS.

### 3.2. Wettabilities of Sponges

**Figure 4:**
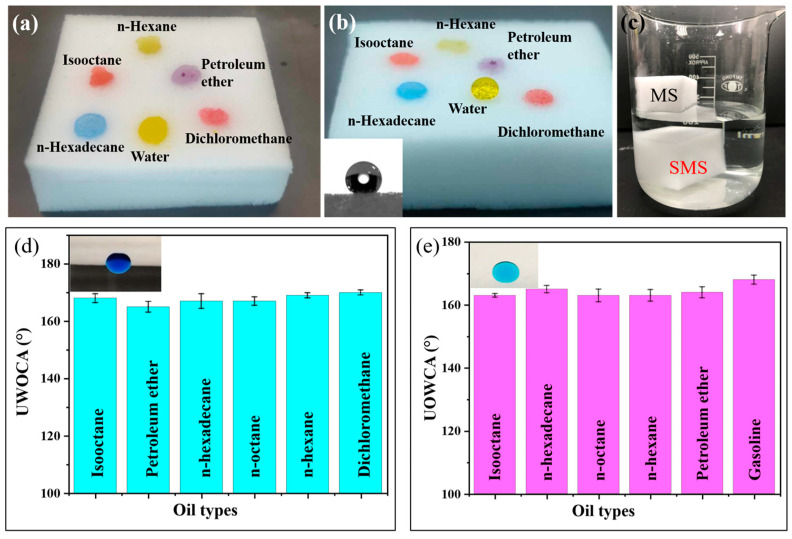
Photographs of the oils and water droplets on (**a**) MS and (**b**) SMS. (**c**) Optical images of MS and SMS in water. (**d**) Underwater oil contact angles and (**e**) underoil water contact angles of SMS.

To assess the wetting characteristics of both the MS and the SMS with respect to oil and water, the dispersion of oil and water droplets was recorded and observed using a charge-coupled device camera system. Both water and different oils (n-hexane, isooctane, n-hexadecane, petroleum ether, and dichloromethane) droplets exhibited spontaneous spreading on the MS surface and were instantly absorbed by the MS. The water contact angle (WCA), as well as the oil contact angles (OCAs) of MS were all close to 0°, indicating that the MS possessed superamphiphilicity in air (Figure 4a). In contrast, SMS showed superhydrophobicity with a WCA of about 160° and superoleophilicity with all OCAs close to 0° (Figure 4b). When the pristine MS and the modified SMS were placed on the water surface, the pristine MS absorbed water and instantaneously sank to the bottom of the glass jar, while the modified MS remained floating on the top of the water (Figure 4c). Our results indicated that the in-air wettability of the MS transformed from superamphiphilic to superhydrophobic/superoleophilic, confirming the successful surface modification. The wettabilities of the SMS in different environments were also investigated, and we found out that the SMS exhibited special, switchable wettabilities. When the SMS was prewetted by water and was forcibly placed underwater, some water turned into its microstructure, and accordingly, in the presence of oil, a composite interface of oil/water/solid was generated. The trapped water diminished the contact surface between the SMS and the oil droplet, resulting in underwater superoleophilicity [51,52]. The underwater oil contact angles (UWOCA) for different kinds of oils on the SMS are shown in Figure 4d. The UWOCA for all oils exceeded 150°, indicating underwater superoleophilicity. A similar situation occurred when we put a water droplet on the SMS under oil. The trapped water decreased the contact area between the SMS and the water droplet, resulting in underoil superoleophilicity. Figure 4e displays that the underoil water contact angles (UOWCA) on the SMS in various oils (including n-hexane, n-octane, isooctane, n-hexadecane, petroleum ether, and dichloromethane) are all higher than 150°, confirming the underoil superhydrophobicity of the SMS. The UWOCAs and UOWCAs were stable for over one hour. It revealed that superwetting properties of the SMS can be switched through the pre-wetting process.

**Figure 5:**
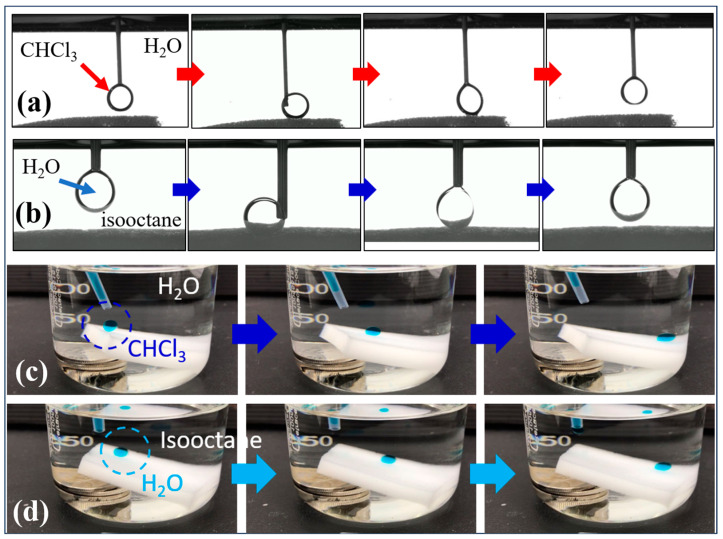
Investigation of (**a**) the underwater anti-oil and (**b**) underoil anti-water characteristics of the SMS. (**c**) Underwater oil droplet rolling test of SMS. (**d**) Underoil water droplet rolling test of SMS.

We then used oil droplets and analyzed their approach, contact, deformation, and departure processes to study the underwater wettabilities of the SMS, such as that of a chloroform droplet suspended on a syringe for the SMS underwater (Figure 5a). The chloroform droplet was promptly and completely withdrawn from the surface of SMS, despite severe deformation occurring in this case. Figure 5b shows a water droplet’s approach, contact, deformation, and departure for the SMS underoil (isooctane). The water droplet was promptly and completely withdrawn from the surface of SMS. We also used oil or water droplet sliding tests to study the wettabilities of the SMS. Figure 5c shows the process of an underwater chloroform droplet rolling off an 18°-tilted SMS. Here, a droplet of chloroform was put on the SMS underwater, and then, after its release from the pipette, the droplet rolled off as soon as it made contact with the SMS. Figure 5d demonstrates the procedure of an underoil (isooctane) water droplet rolling off an 18°-tilted SMS. We put a water droplet on the SMS underoil, and then, after its release from the pipette, the droplet rolled off as soon as it made contact with SMS. These results further confirmed the underwater superoleophobicity and underoil superhydrophobicity of our SMS.

### 3.3. Oil/Water Mixtures Separation Performance of the SMS

**Figure 6:**
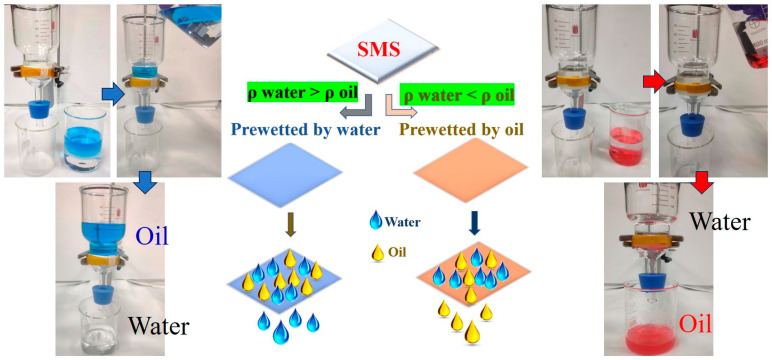
Separation of oil/water mixtures driven by gravity using SMS prewetted with water or oil.

**Figure 7:**
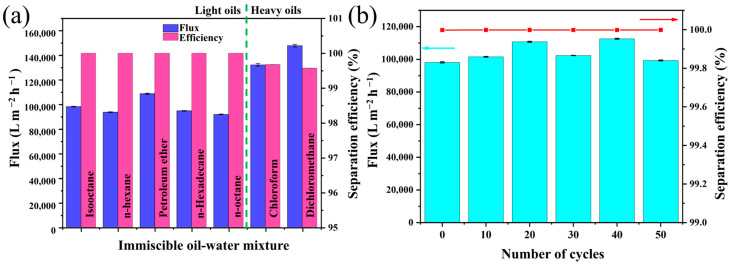
(**a**) Separation performance of the SMS against different oil/water mixtures. (**b**) Cycles of isooctane/water mixture separation flux and efficiency of SMS.

Due to the underwater superoleophobicity and underoil superhydrophobicity of the SMS, it can easily separate both lighter and heavier oil–water mixtures. In our experimental setup, we secured the SMS in the middle of two glass tubes and then prewetted it with water or oil for oil rejection or water rejection separation processes (Figure 6). When the oil density is lower than the water density (ρ_water_ > ρ_oil_), we prewetted the SMS with water under an external pressure to generate the underwater superoleophobic property able to eliminate water from light oil/water mixtures. On the other hand, when the oil density is higher than the water density (ρ_oil_ > ρ_water_), we prewetted the SMS with oil to achieve the underoil superhydrophobic property able to eliminate oil from heavy oil/water mixtures. To facilitate the separation of oil and water, an immiscible oil/water mixture (1:1 volume ratio) was introduced into the separation apparatus and allowed to permeate under gravity. Figure 6 revealed the gravity-driven isooctane/water mixture separation process, where the water penetrated through the SMS (prewetted by water) quickly while the oil was retained above the SMS. However, the water-prewetted SMS is not suitable for treating oil/water mixtures with an oil density greater than water density (e.g., chloroform) because heavy oils could aggregate to form an obstruction layer on the material and prohibit the permeation of water. We solved this problem by prewetting the SMS with heavy oil, thereby producing an underoil superhydrophobic SMS. During the separation test, the chloroform passed through the oil-prewetted SMS briskly, while the water was repelled and remained on the SMS. To further study the separation performance of the SMS, the separation fluxes for different oil/water mixtures were examined. As depicted in Figure 7a, the fluxes of water for different immiscible light oil–water mixtures were demonstrated for isooctane, n-hexane, petroleum ether, n-hexadecane, and n-octane ranging from 98,140, 94,700, 108,600, 93,580, 91,800 L m^−2^ h^−1^, respectively. The separation efficiency for all cases is above 99.99%. On the other hand, the oil fluxes for the heavy oil–water mixture were 132,100 and 147,700 L m^−1^ h^−1^ for chloroform and dichloromethane, respectively, and the separation efficiency for both is greater than 99.56%. These confirm the outstanding performance of the as-prepared SMS for versatile oil/water mixture separations. The reusability of the SMS prewetted by water was evaluated by conducting cyclic tests for separating an isooctane/water mixture. It was revealed that after 50 cycles of separation experiments, the SMS prewetted by water sustained a flux exceeding 98,100 L m^−2^ h^−1^. Moreover, it was found to maintain a separation efficiency higher than 99.99%. This demonstrates the exceptional reusability of the water-prewetted SMS (Figure 7b).

**Figure 8:**
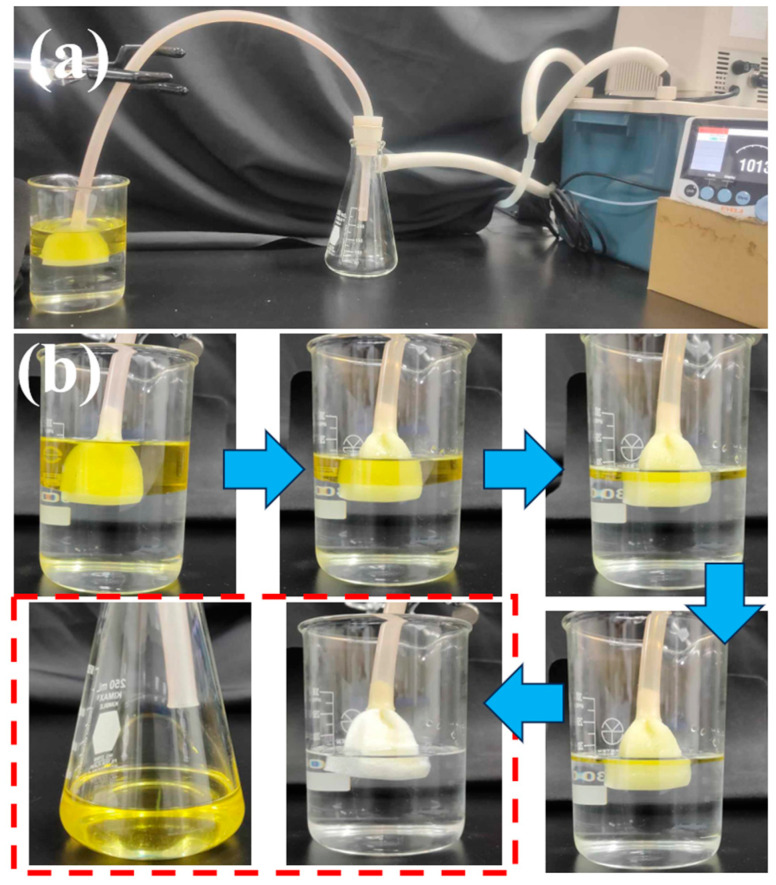
Photographic images of (**a**) the setup for the continuous oil/water separation testing, and (**b**) the stages of the continuous absorption and extraction of an organic solvent from the water surface.

Conventional filtration separation methods are inadequate for effectively addressing large quantities of oil pollutants on water surfaces during oil spill treatment. Therefore, there is a continued necessity to develop innovative technologies that can achieve continuous absorption and elimination of oil contaminants from water surfaces, while possessing an excellent separation capability. In a previous study, we introduced a straightforward setup that involved connecting superhydrophobic porous materials to a vacuum system, enabling one-step removal of substantial quantities of oil pollutants from water surfaces [53]. Herein, we found out that the oil-prewetted SMS can be used for the continuous removal of oil pollutants. A piece of SMS was connected to a tube, prewetted by light oil, and then fixed at the light oil–water interface in a light oil/water mixture (Figure 8a). Figure 8b presents that the SMS was speedily saturated with the oil and completely rejected the water because of its underoil superhydrophobic property. Subsequently, the vacuum system (0.025 bar) was activated to consistently eliminate the oil from the water surface. Eventually, we successfully extracted all the oil, leaving behind only transparent and clean water in the beaker. The collected filtrate oil appeared devoid of any visible water droplets to the naked eye. Notably, upon conducting a continuous separation test of an isooctane/water mixture, high permeance (757,800 L m^−2^ h^−1^ bar^−1^) was observed. In addition, we also checked the purity of the oil filtrate using a Karl Fischer titrator instrument and found that the oil purity exceeded 99.99 wt%, indicating an excellent separation performance of the system. This oil absorption method can be adopted for large-scale removal of oil contaminants from the water surface.

### 3.4. Oil/Water Mixtures Separation Mechanism

**Figure 9:**
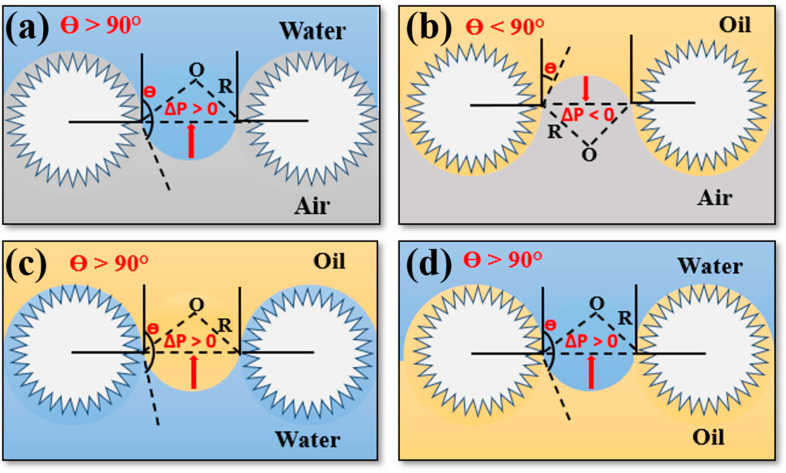
Schematic illustration of the SMS wetting models. (**a**) Water could not permeate through the SMS. (**b**) Oil could permeate through the SMS. (**c**) Oil could not permeate through the SMS prewetted with water. (**d**) Water could not permeate through the SMS prewetted by oil.

To evaluate the oil–water separation mechanism of the SMS, schematic models depicting water and oil wetting were created, as illustrated in Figure 9. To explain the liquid selective properties of the SMS surface, the variation in the breakthrough pressure (ΔP) was demonstrated using the Young–Laplace equation [23,54,55]

(2) $$\Delta P=-\frac{2\gamma_{L}cos\theta }{R}$$

where $\gamma_{L}$ represents the liquid’s surface tension, θ denotes the intrinsic contact angle formed by the liquid on the surface, and R represents the curvature’s pore radius. As can be observed from this equation, it is evident that a material’s wettability has a major impact on the breakthrough pressure. If the θ of a liquid on a surface exceeds 90°, it can endure a certain level of external pressure (Figure 9a). Hence, a given liquid cannot permeate unless external force is applied. Because of the superhydrophobicity of the SMS, we needed to apply external pressure to wet the SMS with water. Conversely, if θ is smaller than 90°, the surface will not be able to sustain a composite interface, allowing the liquid to naturally penetrate through the pores and completely wet the surface. Because of the SMS’ superoleophilicity, the in-air intrinsic contact angle nearly approaches 0°, resulting in ΔP < 0. Hence, a certain oil can promptly penetrate through the SMS and completely wet its surface, as depicted in Figure 9b.

In the water/oil/solid three-phase system, the maximum pressure required to overcome the interfacial tension at the three-phase interface when the surface is prewetted with water or oil is known as the breakthrough pressure. Therefore, the critical intrusion pressure within the interface system of oil–water and solid can be approximated using the formula [55]:

(3) $${\Delta P}_{WO}=-\frac{2\gamma_{L_{1}L_{2}}cos\theta_{OW}}{R}$$

where ${\Delta P}_{WO}$ is the critical intrusion pressure, $\gamma_{L_{1}L_{2}}$ represents the oil/water interfacial tension, and $\theta_{OW}$ corresponds to the water or oil contact angle on the SMS. *R* represents the curvature’s pore radius. Based on this model, when SMS is prewetted either by water or oil, a liquid film could be formed on the material’s surface. Due to this, the UWOCA and UOWCA were observed to exceed 150°, and ${\Delta P}_{WO}$ becomes positive, signifying that the SMS can sustain pressure to some extent. The prewetted SMS allows the wetting phase (water or oil) to quickly permeate through it, while the non-wetting phase is repelled, owing to its underwater superoleophobicity and underoil superhydrophobicity (Figure 9c,d).

### 3.5. Emulsions Separation Performance of the SMS

**Figure 10:**
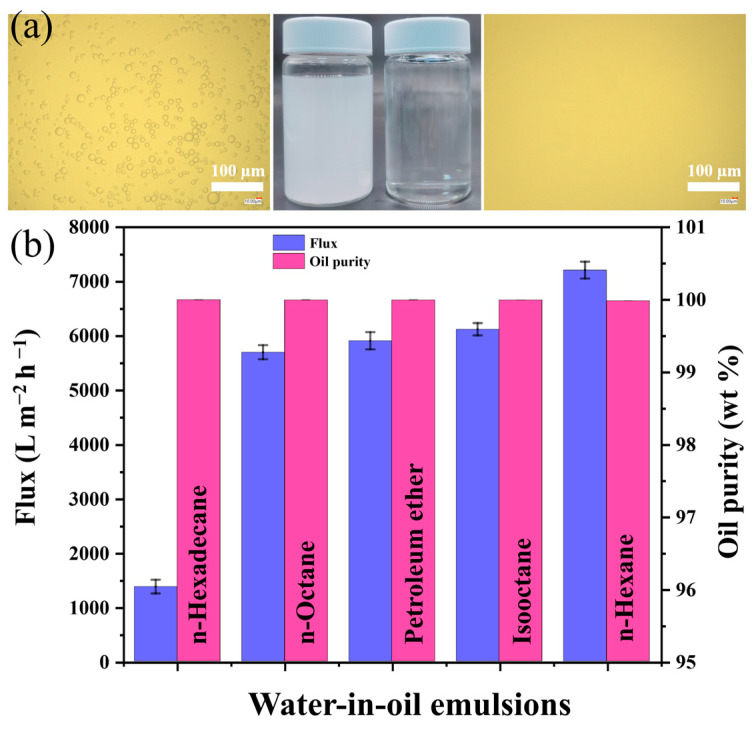
(**a**) Actual photographs and OM images of the SWOE before (**left**) and after (**right**) separation. (**b**) Fluxes and filtrate oil purity of SWOEs.

Wastewater that contains an emulsified oil/water mixture with particles smaller than 20 μm cannot be separated using conventional technologies. Thus, three-dimensional porous materials functionalized with unique wettability have gained interest as a viable option for effective emulsion separation due to their exceptional flexibility [56]. In this study, for the surfactant-stabilized water-in-oil emulsions (SWOEs) separation, a piece of SMS with the desired mass is tightly packed into a funnel with a radius of 0.55 cm and a height of 4 cm. Subsequently, the compressed SMS is prewetted by oil and the SWOE was poured from the top of the funnel allowing the liquid to pass through by gravity. Figure 10a displays the actual photographs, as well as the OM images of the feed SWOE and its corresponding filtrate. The original emulsion contains water droplets with microscale diameters, but after the separation processes these droplets were observed to be removed resulting in a transparent filtrate. These findings indicate that the removal of water from the SWOE was successful. The compressed SMS layer’s density can be adjusted to control the emulsion separation flux and separation performance. As depicted in Figure S1, the optimum compression density with respect to flux and separation efficiency was attained by varying the mass of SMS packed into the funnel. For the water-in-isooctane emulsion separation test, as the density of the SMS increased from 0.039 to 0.163 g cm^−3^, the oil flux was observed to decline from 21,040 to 2130 L m^−2^ h^−1^, and at the same time an enhancement in the emulsion separation performance was found. For the SWOE separation experiments driven by gravity, we selected a compressed SMS having a density of 0.097 g cm^−3^ that exhibited moderate fluxes and separation efficiencies. For the surfactant-stabilized water in n-hexadecane, n-octane, petroleum ether, isooctane, and n-hexane, the separation fluxes were determined to be 1370 ± 126, 5690 ± 130, 5910 ± 160, 6120 ± 110, and 7210 ± 150 L m^−2^ h^−1^, respectively. Correspondingly, the purity of the filtrate oil was tested to evaluate the separation performance. In all cases, it was found that the oil purity of the filtrate was greater than 99.98 wt%, signifying the outstanding separation performance of the compressed SMS (Figure 10b). In comparison to other superwetting materials applied for the separating of SWOEs, SMS exhibits high fluxes and outstanding separation performances (Table S1).

**Figure 11:**
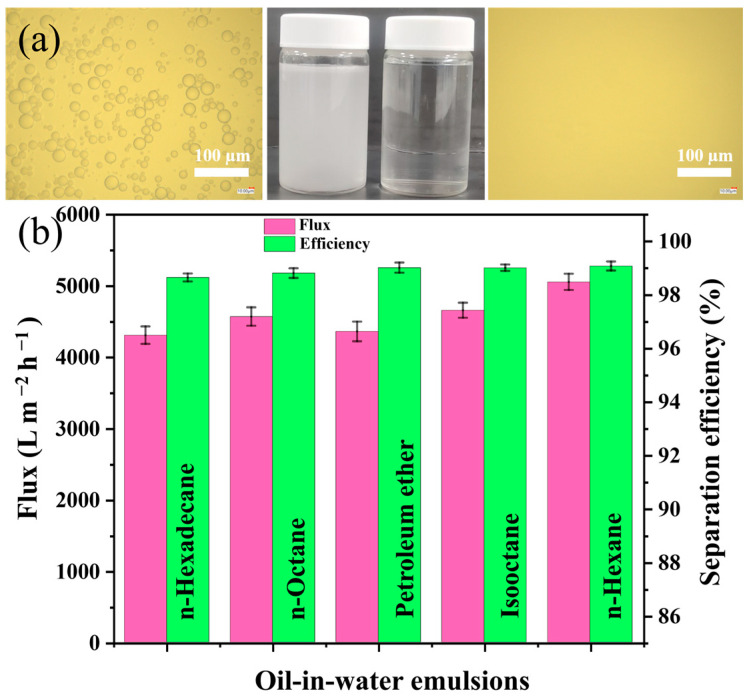
(**a**) Actual photographs and OM images of the SOWE before (**left**) and after (**right**) separation. (**b**) Fluxes and separation efficiencies of SOWEs.

In addition, owing to its switchable superwetting property, the compressed SMS can feasibly be employed in separating surfactant-stabilized oil-in-water emulsions (SOWEs) through a water prewetting process. Figure 11a displays the actual photographs, as well as the OM images of the feed SOWE and its corresponding filtrate. It can be observed that the oil droplets in the feed emulsion can be removed after the separation procedure, yielding a clear filtrate. In the separation test utilizing isooctane-in-water emulsion, as the compressed SMS’ density increased from 0.031 to 0.147 g cm^−3^, the flux correspondingly declined from 17,540 to 1833 L m^−2^ h^−1^, but the separation efficiency increased from 97.75 to 99.06% (Figure S2). To carry out SOWE separation experiments, a density of 0.084 g cm^−3^ was selected as optimal for its moderate flux and reasonable separation performance. The separation fluxes for the surfactant-stabilized n-hexadecane, n-octane, petroleum ether, isooctane, and n-hexane in water, were determined to be 4305 ± 123, 4567 ± 130, 4360 ± 138, 4655 ± 105, and 5054 ± 115 L m^−2^ h^−1^, respectively. For all emulsions, the separation efficiencies were found to exceed 98.84%, signifying an outstanding separation performance (Figure 11b). In comparison to other superwetting materials applied for the separating of SOWEs, SMS exhibits very high fluxes and good separation performances (Table S2).

**Figure 12:**
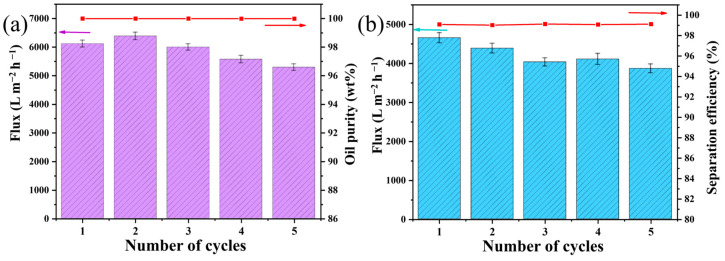
SMS’ recyclability for separating (**a**) SWOEs and (**b**) SOWEs.

SWOEs and SOWEs also underwent cycles of emulsion separation experiments, utilizing isooctane to prepare the emulsions. As shown in Figure 12a,b, even after the fifth cyclic separation test for both SWOEs and SOWEs, the fluxes did not show significant changes. Likewise, the purities in the oil filtrates for all SWOEs exceeded 99.99 wt%, while the separation efficiencies for the SSOIWEs were higher than 99.02%. These findings indicate the remarkable recyclability of the SMS and suggest their feasibility for practical application and long-term usability for emulsion separations.

## 4. Conclusions

The separation of oil/water mixtures and emulsions poses significant challenges, and addressing this issue is crucial as it presents serious environmental concerns. This research tackled and presented the green fabrication method of SMS possessing switchable wettabilities. The as-prepared SMS exhibited superhydrophobicity, superoleophilicity, underwater superoleophobicity, and underoil superhydrophobicity. The SMS was successfully utilized in the separation of both light oil/water and heavy oil/water mixtures, where it exhibited excellent permeation fluxes (reaching 108,600 L m^−2^ h^−1^ for light oil/water mixture, and 147,700 L m^−2^ h^−1^ for heavy oil/water mixture). It also displayed outstanding separation efficiency (exceeding 99.56%). Furthermore, compressed SMS exhibited remarkable gravity-driven emulsion separation capability for both SWOEs and SOWEs, showing high fluxes (reaching 7210 and 5054 L m^−2^ h^−1^, respectively) and outstanding separation performances (with filtrate oil purities for SWOEs exceeding 99.98 wt% and the separation efficiencies for SOWEs exceeded 98.84%). In summary, our research findings illustrate the considerable potential and practicable worth of employing the SMS for real-world use in fuel purification and wastewater treatment applications.

## Data Availability

The data presented in this study are available on request from the corresponding author.

## Supplementary Materials

The following supporting information can be downloaded at: https://www.mdpi.com/article/10.3390/polym16050693/s1, Figure S1: The changes of filtrate flux and separation performance for water-in-isooctane emulsion with increasing the density of the compressed SMS. Figure S2: The changes of filtrate flux and separation efficiency for isooctane-in-water emulsion with increasing the density of the compressed SMS. Table S1: Comparison of various superwetting materials for surfactant stabilized water-in-oil emulsion separations. Table S2: Comparison of various superwetting materials for surfactant stabilized oil-in-water emulsion separations. References [28,29,37,57,58,59,60,61,62,63,64,65] are cited in the supplementary materials.
