# Supplementary material for: An Eco-Friendly Manner to Prepare Superwetting Melamine Sponges with Switchable Wettability for the Separation of Oil/Water Mixtures and Emulsions
Source: Polymers (Basel). 2024 Mar 3;16(5):693. doi: 10.3390/polym16050693 (PMC10934135; doi:10.3390/polym16050693)
Supplement: Supplementary file 1 [file polymers-16-00693-s001.zip › polymers-2899057-supplementary.pdf]

Supporting Information

An Eco-Friendly Manner to Prepare Superwetting Melamine Sponges with Switchable Wettability for The Separation of Oil/water Mixtures and Emulsions

Guyita Berako Belachew[1], Chien-Chieh Hu[1], Yan-Yu Chang[2], Chih-Feng Wang[3]*, Wei-Song Hung[1], Jem-Kun Chen[4], Juin-Yih Lai[1, 5, 6]

1 Graduate Institute of Applied Science and Technology, Advanced Membrane Materials Research Center, National Taiwan University of Science and Technology, Taipei, 106, Taiwan

2 Department of Materials Science and Engineering, I-Shou University, Kaohsiung, 840, Taiwan.

3 Institute of Advanced Semiconductor Packaging and Testing, National Sun Yat-sen University, Kaohsiung, 804, Taiwan

4 Department of Materials Science and Engineering, National Taiwan University of Science and Technology, Taipei, 106, Taiwan

5 R&D Center for Membrane Technology, Chung Yuan Christian University, Taoyuan, 320, Taiwan

6 Department of Chemical Engineering and Materials Science, Yuan Ze University, Taoyuan,320, Taiwan

*To whom all correspondence should be addressed

E-mail: cfwang@mail.nsysu.edu.tw

Tel: 886-7-5252000-6610

Materials

Commercial melamine sponge was purchased from BASF. HCl (37%) and Chloroform (99%) were purchased from Fluka. Sodium Laurate (>97%) was purchased from Tokyo Chemical Industry Co., Ltd.. Petroleum ether (60/80) and n-hexadecane (99%) were purchased from Alfa Aesar. Span 80, tween 80, and n-octane (98%) were purchased from Acros Organics. Isooctane (99.7%), and n-hexane (95%) were purchased from TEDIA Company Inc.

Instruments and characterization

The surface morphology was evaluated by Scanning electron microscopy (SEM; JEOLJSM-6500, JEOL. Ltd.). Attenuated total reflection Fourier transform infrared spectra (ATR-FTIR, PerkinElmer, Inc.) was used to record the surface composition of the pristine and superwetting MS. The static contact angle on the pristine and superwetting MS was evaluated through a Magic Droplet-100 contact angle goniometer (Sindatek Instruments Co., Ltd.) by injecting a 5 μL liquid droplet. To analyze water content in the permeate, a titrator Compact C10SX Coulometric Karl Fischer moisture titrator (Mettler-Toledo PacRim AG) was used. A total organic carbon analyzer (Vario TOC, Elementar) was used to measure the oil content in the filtrate. Optical microscopy images were characterized by a VHX-7000 instrument (Keyence Corp.).

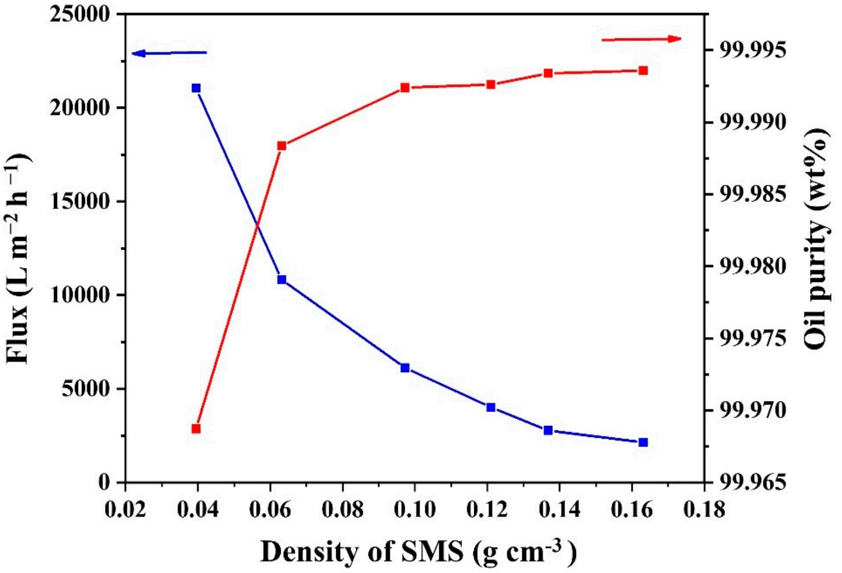

Figure S1. The changes of filtrate flux and separation performance for water-in-isooctane emulsion with increasing the density of the compressed SMS layer.

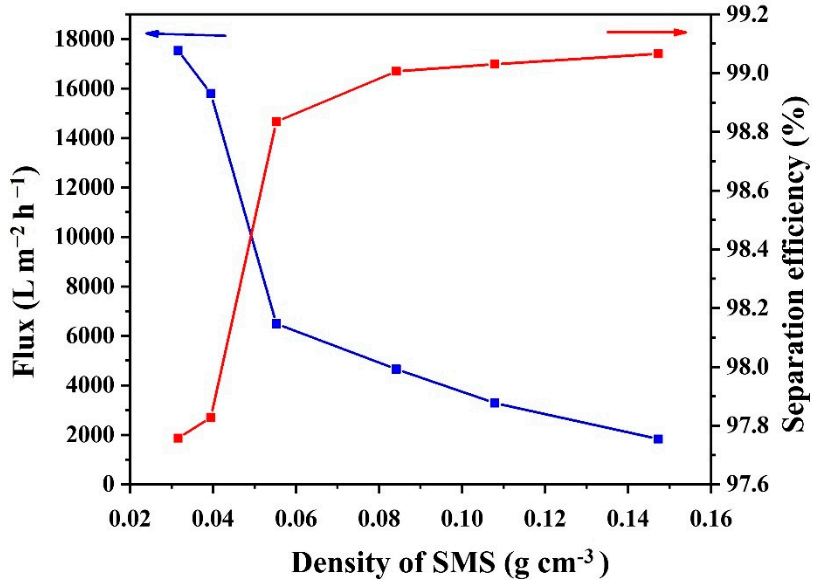

Figure S2. The changes of filtrate flux and separation efficiency for isooctane-in-water emulsion with increasing the density of the compressed SMS.

Table S1. Comparison of various superwetting materials for surfactant stabilized water-in-oil emulsion separations.

Materials	Water-in-oil emulsion separation		Reference
	Flux (L m^{-2} h^{-1})	Oil purity (wt%)	
FPBZ/SiO_2@Melamin sponge	1500	>97.6	**[S1]**
PVPh/PBO coated raw cotton	10,400	>99.98	**[S2]**
PBZ-SiO_2-MS	1300	>99.9	**[S3]**
3D Composite PXSNF	6200	>99.96	**[S4]**
Dually prewetted carbon black membrane (DCBM)	8500	>99.6	**[S5]**
Superhydrophobic copper foam	6560	>99.89	**[S6]**
Superwetting melamine sponge (SMS)	7210	>99.98	This work

Table S2. Comparison of various superwetting materials for surfactant stabilized oil-in-water emulsion separations.

Materials	Oil-in-water emulsion separation		Reference
	Flux (L m^{-2} h^{-1})	Efficiency (%)	
GO/g-C_3N_4/TiO_2 Melamine foam	1910.8	>99	**[S7]**
Superhydrophilic Ti foam	1033	>98	**[S8]**
Biomimetic hydrophilic membrane (MF-D/T-2/8)	2100	>99.6	**[S9]**
WBG (waste bricks) layer	1079	>98.4	**[S10]**
Silica-decorated microfiltration membranes	1431	>99	**[S11]**
Waste-coal fly ash (CFA)	1050	>92	**[S12]**
Superwetting melamine sponge (SMS)	5054	>98.84	This work

References

[S1] Ge J., Wang F., Yin X., Yu J., Ding B. Polybenzoxazine-functionalized melamine sponges with enhanced selective capillarity for efficient oil spill cleanup, ACS Appl. Mater. Interfaces 10(46) (2018) 40274-40285.

[S2] Ejeta D.D., Wang C.-F., Kuo S.-W., Chen J.-K., Tsai H.-C., Hung W.-S., Hu C.-C., Lai J.-Y. Preparation of superhydrophobic and superoleophilic cotton-based material for extremely high flux water-in-oil emulsion separation, Chem. Eng. J. 402 (2020) 126289.

[S3] Peng Y., Liu Y., Dai J., Cao L., Liu X. A sustainable strategy for remediation of oily sewage: Clean and safe, Sep. Purif. Technol. 240 (2020) 116592.

[S4] Chu Z., Seeger S., Multifunctional hybrid porous micro-/nanocomposite materials, Adv. Mater 27(47) (2015) 7775-7781.

[S5] Cao G., Wang Y., Wang C., Ho S.-H. A dually prewetted membrane for continuous filtration of water-in-light oil, oil-in-water, and water-in-heavy oil multiphase emulsion mixtures, J. Mater. Chem. A 7(18) (2019) 11305-11313.

[S6] Wang Z., Xiao C., Wu Z., Wang Y., Du X., Kong W., Pan D., Guan G., Hao X. A novel 3D porous modified material with cage-like structure: fabrication and its demulsification effect for efficient oil/water separation, J. Mater. Chem. A 5(12) (2017) 5895-5904.

[S7] Zhan B., Liu Y., Zhou W.-T., Li S.-Y., Chen Z.-B., Stegmaier T., Aliabadi M., Han Z.-W., Ren L.-Q. Multifunctional 3D GO/g-C3N4/TiO2 foam for oil-water separation and dye adsorption, Appl. Surf. Sci. 541 (2021) 148638.

[S8] Zhang L., Yang X., Jiang B., Sun Y., Gong Z., Zhang N., Hou S., Li J., Yang N. Superhydrophilic and underwater superoleophobic Ti foam with robust nanoarray structures of TiO2 for effective oil-in-water emulsion separation, Sep. Purif. Technol. 252 (2020) 117437.

[S9] Yang X., Yan L., Wu Y., Liu Y., Shao L. Biomimetic hydrophilization engineering on membrane surface for highly-efficient water purification, J. Membr. Sci. 589 (2019) 117223.

[S10] Li Z., Wu J., Yue X., Qiu F., Yang D., Zhang T., Study on the application of waste bricks in emulsified oil-water separation, J. Clean. Prod. 251 (2020) 119609.

[S11] Yang H.-C., Pi J.-K., Liao K.-J., Huang H., Wu Q.-Y., Huang X.-J., Xu Z.-K. Silica-decorated polypropylene microfiltration membranes with a mussel-inspired intermediate layer for oil-in-water emulsion separation, ACS Appl. Mater. Interfaces 6(15) (2014) 12566-12572.

[S12] Li Z., Qian W., Chen Y., Xu P., Li J., Yang J. A new treasure in industrial solid waste—Coal fly ash for effective oil/water separation, J Taiwan Inst Chem Eng 118 (2021) 196-203.
